# Quantum speed limit and a signal of quantum criticality

**DOI:** 10.1038/srep19308

**Published:** 2016-01-19

**Authors:** Yong-Bo Wei, Jian Zou, Zhao-Ming Wang, Bin Shao

**Affiliations:** 1 School of Physics, Beijing Institute of Technology, Beijing 100081, China; 2 Department of Physics, Ocean University of China, Qingdao 266100, China

## Abstract

We study the quantum speed limit time (QSLT) of a coupled system consisting of a central spin and its surrounding environment, and the environment is described by a general XY spin-chain model. For initial pure state, we find that the local anomalous enhancement of the QSLT occurs near the critical point. In addition, we investigate the QSLT for arbitrary time-evolution state in the whole dynamics process and find that the QSLT will decay monotonously and rapidly at a large size of environment near the quantum critical point. These anomalous behaviors in the critical vicinity of XY spin-chain environment can be used to indicate the quantum phase transition point. Especially for the XX spin-chain environment, we find that the QSLT displays a sudden transition from discontinuous segmented values to a steady value at the critical point. In this case, the non-Makovianity and the Loschmidt echo are incapable of signaling the critical value of the transverse field, while the QSLT can still witness the quantum phase transition. So, the QSLT provides a further insight and sharper identification of quantum criticality.

Classical phase transition occurs when a physical system reaches a state which is characterized by some macroscopic order parameter, such as a critical temperature. It is driven by a competition between the energy of a system and the entropy of its thermal fluctuations. In contrast, quantum systems have fluctuations driven by the Heisenberg uncertainty principle even in the ground state, and these can drive interesting phase transitions at absolute zero temperature. Such a quantum phase transition (QPT) can be accessed by varying external parameters or coupling constant, and is certainly one of the major interests in condensed matter physics^1^.

Recently, the QPT has drawn considerable interest in fields of quantum information science^2^,^3^,^4^,^5^,^6^,^7^. Many quantities have been found to capture the ground state singularities associated with a QPT, such as entanglement, geometric Berry phase, and non-Markovianity^4^,^5^,^6^,^7^,^8^,^9^,^10^,^11^. Some investigations showed that the time-energy uncertainty relation in quantum mechanics establishes a fundamental bound for the evolution time *τ* between two states of a given closed system, *τ* ≥ *max*{*πħ*/2Δ*E*, *πħ*/2*E*}^12^,^13^. This minimal time that a system needs to evolve from an initial state to an orthogonal target state is defined as the quantum speed limit time (QSLT), which can be used to characterize the maximal evolution speed^12^,^13^. The manifold applications of these limits have been shown in many fields, such as quantum communication^14^, quantum metrology^15^, the formulation of computational limits of physical systems^16^, as well as the quantum optimal control algorithms^17^. Generalization of this fundamental concept to the real-time evolution of an open system has been done recently^18^,^19^,^20^,^21^,^22^,^23^,^24^,^25^,^26^,^27^,^28^,^29^.

As a quantum critical phenomenon, QPT happens at zero temperature. It is only driven by quantum fluctuation, and the uncertainty relation lies at the heart of various QPT phenomena. The QSLT determines the theoretical upper bound on the speed of evolution. It is a generalization of the Heisenberg uncertainty relation of energy and time^12^,^13^. In single qubit open systems, it was found that the QSLT is susceptive to the variation of environment parameter and entanglement of subsystem, and the memory effect of environment plays a decisive role in its reduction^23^,^24^. So it is very intriguing to investigate whether the QSLT in an open system can be extended to the macroscopic regions to capture the QPT, as correlations and geometric Berry phase are?

Here, we connect the QSLT with quantum criticality by exploring the QSLT of an open system near its critical point. The whole system constitutes of a central spin and spin environment modeled by an XY spin chain. We find that the QSLT is anomalous at the critical point. Interestingly, with the increasing of driving time (i.e., the actual evolution time) or the size of the chain, the critical characteristic of QSLT becomes more remarkable at QPT point.

In addition, we investigate the QSLT for different magnetic field strength in the whole dynamics process of central spin. We find that the decay of QSLT is dramatically accelerated in the vicinity of the quantum critical point of spin environment. By utilizing the relationship between the Loschmidt echo (LE) and entanglement, we elucidate the speed-up mechanism of entanglement. Our results show that the key ingredients of quantum criticality are present in the QSLT of the central spin.

## Results

### The model

The system under consideration is a central spin coupled to a spin-1/2 XY chain, which consists of *N* spins with nearest neighbor interactions and an external magnetic field^8^,^10^,^11^. The total Hamiltonian is


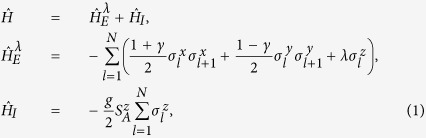


where 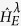 represents the self-Hamiltonian of the spin-chain environment and 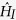 denotes the interaction between the central spin and the environment, with *g* denoting their coupling strength. The spin operators 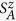 and 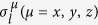 are used to describe the central spin and the surrounding chain, respectively. We assume periodic boundary condition and take *ħ* = 1 for simplicity. The parameter *λ* characterizes the strength of the transverse magnetic field applied in the *z* direction, and *γ* is anisotropic parameter. *γ* = 1 corresponds with the Ising model, whereas for *γ* = 0 it is the XX model. For the quantum criticality in the XY model, there are two universality classes for the parameter *γ*. The critical features are characterized in terms of a critical exponent *ν* defined by *ξ* ~ |*λ* − *λ*_*c*_|^−*ν*^, and *ξ* represents the correlation length. For any value of *γ*, quantum criticality occurs at the critical magnetic field *λ*_*c*_ = 1. For 0 < *γ* ≤ 1, the model belongs to the Ising universality class characterized by the critical exponent *ν* = 1, which is in the Ising-like phase; while for *γ* = 0 the model belongs to the XX universality class with *ν* = 1/2, corresponding to the spin-fluid phase^1^. The density matrix of central spin for arbitrary time *t* can be obtained analytically (see Methods).

### Central system quantum speed limit and quantum critical phenomenon

In the following, we use a unified lower bound for the minimal evolution time of an open quantum system. Using the Bures angle 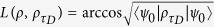, the intrinsic speed has been derived for the evolution between the initial pure state 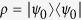 and the final state 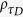, here *τ*_*D*_ is the driving time^23^. The quantum system is governed by the master equation *ρ*_*t*_ = *L*_*t*_(*ρ*_*t*_), and *L*_*t*_ is the positive generator of the dynamical semigroup Λ_*t*_ = exp(*L*_*t*_). A unified expression for the QSLT of arbitrary initially pure states in open systems can be written as,


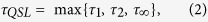


with


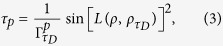


where 
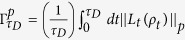
. Different *p* correspond with the operator norm (*p* = ∞), the trace norm (*p* = 1) and the Hilbert-Schmidt norm (*p* = 2), respectively. 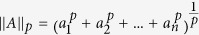 denotes the *p*-norm of operator A, and *a*_1_,..., *a*_*n*_ are the singular values of operator A.

Suppose that the initial state of the central spin is set to be 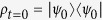 with 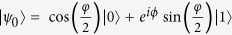 (*φ* ∈ [0, *π*], *ϕ* ∈ [0, 2*π*]), with the central spin up 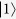 and down 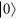. For our model, the QSLT of the central qubit can be described by Margoius-Levitin (ML) type bound based on the operator norm, which provides the sharpest bound^23^. For a driving time *τ*_*D*_, the QSLT from *ρ*_0_ to 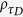 can be expressed as


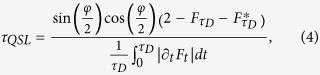


where *F* is the decoherent factor of the central spin density matrix and its norm gives a quantity known as the LE (or fidelity): *L*(*t*) = |*F*(*t*)|^2^. 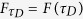 is the decoherent factor at *t* = *τ*_*D*_. Note that *τ*_*QSL*_ depends on the dephasing rate of the environment and the driving time *τ*_*D*_. In the following, we will show that the QSLT also relates to the parameters of the environment, such as the magnetic field *λ*, the anisotropy parameter *γ*, the interaction strength *g*, and the environment size *N*, *etc*. Without loss of generality, we set the parameter *φ* = *π*/2.

**Figure 1:**
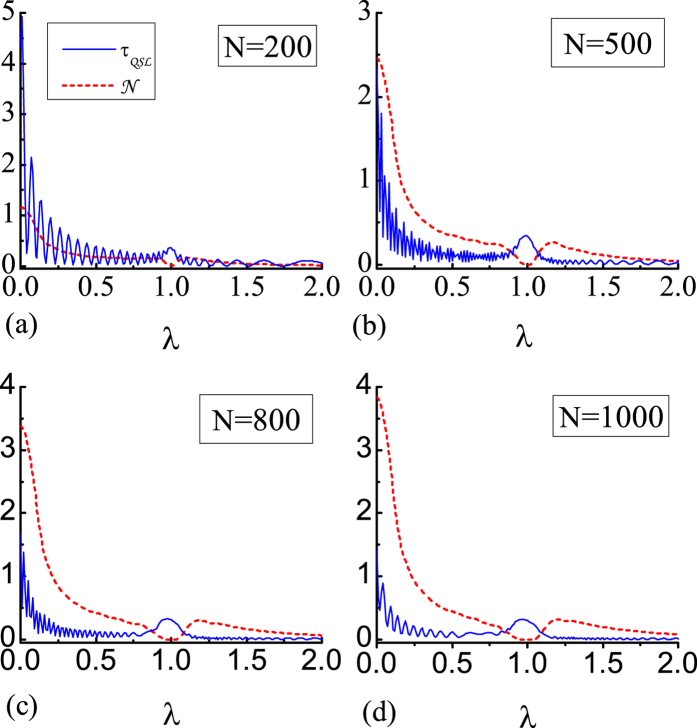
The QSLT *τ*_*QSL*_ and non-Markovianity N as functions of the external magnetic field strength *λ*. The curves in (**a**–**d**) correspond to different environment sizes *N* = 200, 500, 800, 1000. Here we set weak couple *g* = 0.05, *γ* = 1 and the driving time *τ*_*D*_ = 10. There are clear anomalies for the QSLT (blue solid line) and non-Markovianity 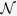 (red dashed line) near the critical point *λ*_*c*_ = 1.

In order to reveal the relationship between the QSLT and the QPT, in Fig. 1 we plot the QSLT as a function of magnetic field *λ* in the weak coupling regime. For simplicity, we set *γ* = 1, in this case the spin chain becomes the Ising model. Two features are notable: (i) the local anomalous enhancement of the QSLT near the critical point *λ*_*c*_ = 1. Figure 1 shows that when *λ* approaches *λ*_*c*_, *τ*_*QSL*_ has a local maximum at *λ*_*c*_. (ii) two oscillating regions divided by *λ*_*c*_. In Fig. 1, *τ*_*QSL*_ oscillates strongly far from the critical point, especially for small values of the magnetic field. When *λ* ∈ (0, 1), *τ*_*QSL*_ oscillates drastically with the increasing of *λ*, while for *λ* ∈ (1, 2) the oscillation becomes weaker. This oscillatory behavior depends on the value of *λ*, indicating that the system is susceptive to the perturbation caused by the surrounding environment. Thus, the anomalous enhancement of QSLT near *λ*_*c*_ can also be used as a witness of QPT. In addition, from Fig. 1(a–d), the evolution of central spin can be accelerated with increasing *N*.

The critical behavior of QSLT can be explained by non-Markovianity. Recent investigations have shown that the non-Markovianity and the associated information backflow from the reservoir can speed up quantum evolution which corresponds to smaller QSLT^23^,^24^. The definition of the non-Markovianility is shown in Methods.

In Fig. 1 we also plot the non-Markovianity 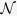 (red dashed line) as a function of external magnetic field *λ*. Obviously, the qubit dynamics exactly becomes Markovian at the critical point, while outside the critical point the non-Markovian effects always exist. Since the environment is non-Markovian except for the critical point, the QSLT near *λ*_*c*_ is longer. When 0 < *λ* < 1, strong non-Markovianty exhibits while *λ* > 1 corresponds to the weak non-Markovianty. The non-Markovianty indicates that the information exchange between system and environment, particularly for small *λ*^9^. Then stronger oscillation of QSLT in small *λ* exhibits. The non-Markovianity is also dependent on the size of environment. The larger number *N* is, the larger value of 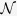 will be.

**Figure 2:**
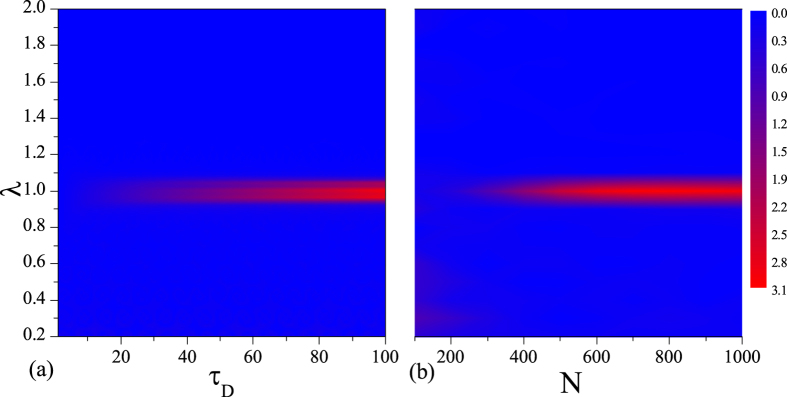
(**a**) The QSLT *τ*_*QSL*_ as a function of driving time *τ*_*D*_ and external magnetic field strength *λ*, with the environment size *N* = 1000. (**b**) The QSLT *τ*_*QSL*_ as a function of environment size *N* and field strength *λ*, with the driving time *τ*_*D*_ = 100. Here, we set *g* = 0.05, *γ* = 1 in both (**a**,**b**). There are clear anomalies near the critical point *λ*_*c*_ = 1.

From Eq. (4) and Fig. 1, we can see that the QSLT depends on the driving time *τ*_*D*_ and the size *N*. We plot the QSLT as a function *λ* and *τ*_*D*_ in Fig. 2(a) and *N* in Fig. 2(b), respectively. In Fig. 2(a) a highlighted critical characteristic of spin environment demonstrates near the critical point *λ*_*c*_ with increasing *τ*_*D*_. The longer of the driving time *τ*_*D*_ is, the larger value of QSLT at critical point will be. Similarly, for fixed driving time *τ*_*D*_, Fig. 2(b) shows that by increasing *N*, the QSLT is increased in the vicinity of QPT point. Critical singularity becomes more prominent for bigger *N* and *τ*_*D*_. In a word, the above results show that the QSLT captures the characteristic of QPT, and can be exploited as a tool to detect criticality even in small size of spin environment.

So far we only consider the Ising model (*γ* = 1). For the XY model, there are two distinct critical regions in the parameter space: the segment (*γ*, *λ*) = (0,(0, 1)) for the XX chain and the critical line *λ*_*c*_ = 1 for the whole family of the XY model (including *γ* = 1)^1^,^7^. For the XX chain (*γ* = 0), the LE equals to unity (|*F*(*t*)| = 1) during the time evolution, regardless of the variation of *λ*^10^. As a consequence, the off-diagonal terms can be expressed as *ρ*_01_(*t*) = [*ρ*_*S*_(0)]_01_*e*^*iθ*(*t*)^, i.e., only the phase factor of *ρ*_01_ evolves from the initial state *θ*(0) to the final state *θ*(*t*).

**Figure 3:**
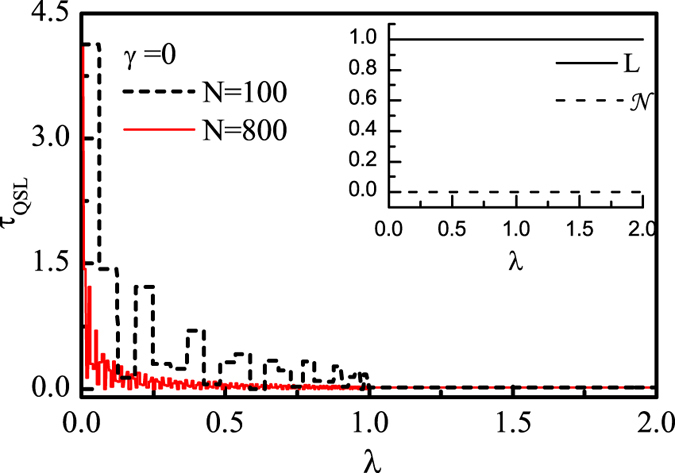
The QSLT as a function of magnetic field strength *λ* for a XX spin-chain environment (*γ* = 0), with the size *N* = 100,800, *g* = 0.05 and the driving time *τ*_*D*_ = 10. Inset shows that when *γ* = 0, the Loschmidt echo *L* is always unit and the non-Markovianity 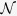 is always zero, regardless of what *N*, *λ* and *g* are.

In Fig. 3 we plot the QSLT as a function of magnetic field strength *λ* for anisotropic parameter *γ* = 0. A notable discontinuity of sudden transition from oscillatory value to a steady value occurs at *λ*_*c*_. Interestingly, we find that there exist discontinuous platforms of QSLT especially for small size *N* (for example *N* = 100 in Fig. 3). This behavior is similar to the critical property of the ground-state Berry phase for the central spin^10^. After passing through the critical point *λ*_*c*_ = 1, the QSLT keeps a steady value which turns out to be determined by the system-environment coupling parameter *g* and the size *N*, as well as the driving time *τ*_*D*_. For bigger *N* (for example *N* = 800 in Fig. 3), the oscillation becomes more drastic and the platforms become narrower. The segmented behavior of QSLT for finite size is a unique feature of the XX model, and it will be completely washed out by increasing *γ*.

As we have known, the Loschmidt echo *L*(*t*) and the non-Markovianity 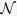 can indicate the critical point of XY spin-chain environment with finite size *N*^8^,^9^. For the XX chain (*γ* = 0), |*F*(*t*)| is always equal to unity, regardless of the variation of *λ* and the size of the spin chain. In Eq. (13), the non-Markovianility depends on the rate of change of the trace distance ∂_*t*_*D*_*t*_ based on the optimal state pairs, where ∂_*t*_*D*_*t*_ = ∂_*t*_|*F*_*t*_|. Hence, as we see in inset of Fig. 3, 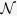 is zero and *L*(*t*) is unit always. In this case, both methods are incapable of signalling the QPT of spin environment, while the variation of QSLT with *λ* can still reflect the quantum criticality.

**Figure 4:**
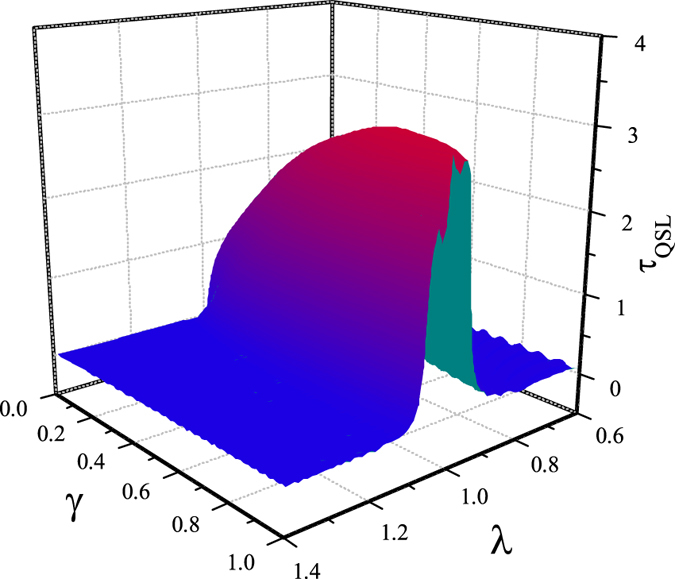
The QSLT as a function of spin-chain parameters *γ* and *λ*, with *N* = 1000, and the driving time *τ*_*D*_ = 100. Here we set weak coupling *g* = 0.05.

Next we plot the QSLT as a function of parameters *γ* and *λ* in Fig. 4. It shows that with the increasing of the parameter *γ*, the local maximum of QSLT becomes more pronounced, and its critical property becomes more noticeable.

### The QSLT of the whole dynamical process and quantum criticality

In the following, we focus on the inherent relation between the QSLT and QPT from the perspective of the whole dynamics process. Eqs. (2) and (3) describe the intrinsic speed for the evolution between a pure state 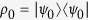 and final target state 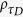. They are not suitable for mixed initial states. Fortunately, ref. 25 gives another unified expression for the QSLT of arbitrary initially mixed states *ρ*_*t*_ in open systems. By using the so-called relative purity 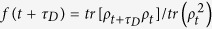, another unified expression for the QSL time of arbitrary initial mixed state *ρ*_*t*_ in open systems has been derived^25^,


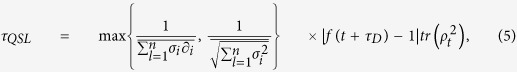


where *σ*_*i*_ is the singular value of *L*_*t*_(*ρ*_*t*_) and *∂*_*i*_ is the singular value of the mixed initial state *ρ*_*t*_. It can be used to demonstrate the quantum evolution speed from a mixed initial state *ρ*(*t*) to another *ρ*(*t* + *τ*_*D*_) for the driving time *τ*_*D*_. Here, we consider the system evolution starting from initial state 
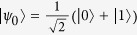
 in our model. Under the action of spin-chain environment, the system evolves to *ρ*_*t*_ as time increases. Hence, using Eq. (5) the QSLT for an arbitrary time-evolution state *ρ*_*t*_ can be calculated as


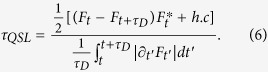


It can be used to study the variation of quantum evolution speed in the whole dynamic process. In the following, we will use this expression to investigate the relation between the QSLT and quantum criticality.

**Figure 5:**
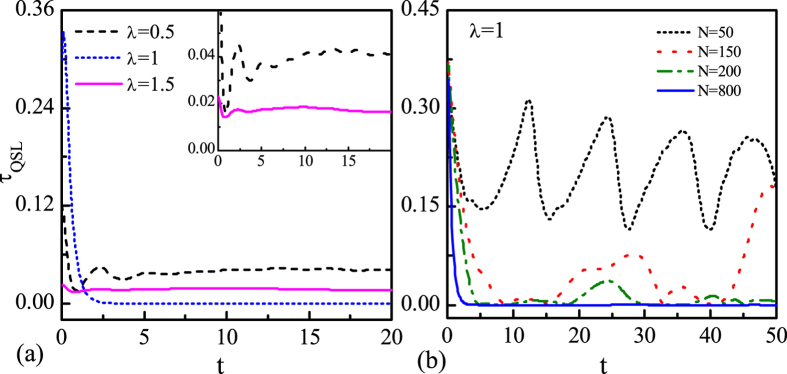
(**a**) The QSLT *τ*_*QSL*_ as a function of the time *t* with different magnetic field strength *λ*, and *N* = 1000. Inset: the oscillation behaviors exhibit for *λ* = 0.5 (dashed line) and *λ* = 1.5 (solid line). (**b**) The QSLT *τ*_*QSL*_ as a function of time *t* with different *N* at critical point *λ* = 1. Here we set weak coupling *g* = 0.05, *γ* = 1, and the driving time *τ*_*D*_ = 10.

The QSLT for different *λ* is plotted in Fig. 5(a) in the whole evolution process. One can see that at *t* = 0, the longest *τ*_*QSL*_ corresponds to the critical point *λ* = 1. When *λ* = 1, *τ*_*QSL*_ decays monotonically to zero without any revival while for other *λ*, there exists oscillation behavior. Figure 5(b) demonstrates the influence of size *N* on the decay behavior of QSLT at *λ* = *λ*_*c*_. At this critical point, the QSLT decays and revives as time increases. As expected, the QSLT decays more rapidly by increasing the size *N*. The singular behavior of QPT at *λ* = *λ*_*c*_ exhibits the hypersensitivity of the ground states of the surrounding system with respect to the perturbation coupling imposed by the central system^8^. This quantum criticality can be reflected by the QSLT. The decays and revivals may serve as a good witness of QPT in the case of finite size *N*.

The critical behavior can be physically explained as follows. The qubit dynamics becomes exactly Markovian at the critical point. It leads to a longer QSLT at the initial time than that out of the critical point *λ* = 1. It is well known that entanglement is a resource that can enhance the evolution speed^30^,^31^,^32^,^33^. Recent research has shown that the entanglement between subsystems in multiqubit open systems is able to reduce the QSLT, i.e., accelerates quantum evolution^26^. In our model the entanglement between system and environment causes the speed-up evolution of the central spin. In the whole dynamics process, the entanglement is enhanced with the continuous decay of the LE^8^, and consequently results in the accelerating dynamical evolution of the system (or equivalently, the decrease of QSLT). Especially, at QPT point the entanglement monotonously increases and the maximal entanglement can be obtained in the thermodynamic limit. Thus, in the long time limit the speed-up effect of entanglement leads to the monotonous decrease of QSLT and faster speed-up evolution process.

**Figure 6:**
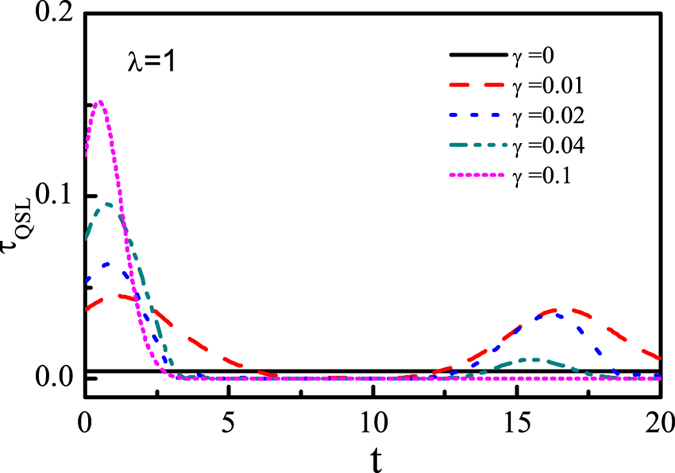
For the whole dynamics process, the QSLT as a function of the time *t* for different anisotropic parameter *γ* at critical point *λ* = 1, with *N* = 800. Here we set weak coupling *g* = 0.05, and the driving time *τ*_*D*_ = 10.

At last, we investigate the influence of the anisotropy parameter *γ* on the QSLT from the aspect of the whole dynamical process. Figure 6 shows that the QSLT as a function of time *t* for different values of *γ* in the quantum critical point *λ*_*c*_ = 1. Obviously, *γ* plays a significant role in the QSLT at the initial time and causes subsequently decay as time increases. When *γ* = 0, *τ*_*QSL*_ always equals to a constant (black solid line) regardless of the time, that is, the speed-up evolution does not exist. The reason is that when *γ* = 0 the LE remains unity during the time evolution, indicating that there is no entanglement generation between the central spin and environment. With *γ* away from zero, the QSLT at the initial time will be enhanced. Increasing the value of *γ* further will result in the decreasing of the oscillations amplitude and a complete decay without prominent revivals. Note that there are some time windows in which the QSLT equals zero (e.g., a time window from *t* = 5 to *t* = 12). The reason is that the states of system reach completely mixed states during these time intervals. Hence the QSLT is zero during the corresponding time window. Therefore, the QSLT at the initial time and its decay rate can be tuned by the anisotropy parameter *γ*.

## Discussion

We have analyzed the behavior of the QSLT in a system consisting of a central spin and its surrounding environment and established a connection between the QSLT and QPT in a general many-body system. The exact expressions of the QSLT have been obtained. We find that the QSLT has some strong imprint of the QPT for the XY model, even for a finite-sized environment. The QSLT shows some noticeable anomalous behaviors near the critical point. These properties are attributed to the non-Markovian or Markovian nature of the environment. With the increasing of the driving time, the size of chain or the anisotropy parameter, the critical characteristic of the QSLT becomes more prominent at QPT critical point. By the heuristic analysis and the numerical calculations, we find that the QSLT provides a further insight and sharper identification of quantum criticality. Especially for the XX spin-chain environment, the 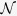 and the LE are incapable of signalling the critical value, while the QSLT still can witness the QPT of spin environment.

Furthermore, we have investigated the QSLT for different magnetic field strength in the whole dynamics process and find that the quantum critical behavior of spin-chain environment causes the monotonous and rapid decay of QSLT at the large size of environment, while out of the critical point the QSLT displays oscillation behavior. At QPT point the entanglement between system and environment causes faster speed-up evolution process. Then the QSLT can also be used to reveal the quantum criticality in the perspective of dynamics process.

Indeed, our result shows that the QSLT has the highlight property of being able to signal the critical value of the transverse field even away from the thermodynamic limit where the quantum phase transition truly takes place. Generalizations of these results to a wide variety of critical phenomena and their relation to the critical exponents are a promising and challenging question that deserves extensive investigation in the future.

## Methods

### The density matrix of central spin for arbitrary time *t*

Following refs 8 and 10, we can rewrite the total Hamiltonian Eq. (1) as


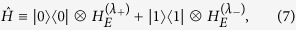


where 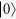 and 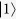 denote the eigenstates of *σ*^*z*^ with eigenvalues of ±1. 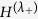 and 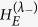 are the corresponding effective Hamiltonians of the spin chain. We use parameters *λ*_+_ = *λ* + *g* and *λ*_−_ = *λ* − *g* to denote the intensity of the magnetic field for two effective Hamiltonians 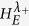 and 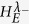, which are defined as 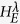 in Eq.(1) by replacing *λ* with *λ*_+_ and *λ*_−_, respectively. 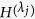 (*j* = +, −) can be diagonalized by standard procedure^1^, i.e., by using the Jordan-Wigner transform followed by a Fourier transform and finally a Bogoliubov rotation. The diagonalized form can be expressed as:


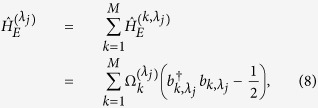


where the energy spectrum is given by


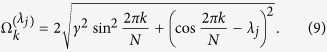


Suppose the central system and the chain environment are initially uncorrelated. The initial density matrix of the composite system can be described by the product state as *ρ*(0) = *ρ*_*S*_(0) ⊗ *ρ*_*E*_(0), where 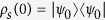 and 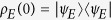 are the initial density matrix of the central system and the environment respectively. We suppose that the spin chain is initially in the ground state. The evolved density matrix of the total system for *t* > 0 is *ρ*(*t*) = *U*(*t*)*ρ*(0)*U*^†^(*t*), where *U*(*t*) is the time evolution operator. It can be rewritten as


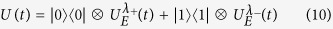


where 
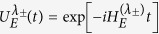
 is the effective time evolution operator. The reduced density matrix of the central system reads^8^,^10^


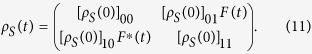


Eq. (11) reveals that the spin chain only modulates the off-diagonal terms of *ρ*_*S*_(*t*) through the decoherence factor 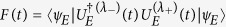 and its norm known as the LE has been found to capture the ground state singularities associated with a QPT^8^,^10^.

### Measure of non-Markovianity

The measure of non-Markovianity we employ here has been defined by Breuer *et al.*, which is based on the information flow between a system and its environment. Considering a quantum process, the measure is defined as the total backflow of information^34^


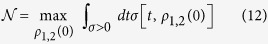


with the maximization over all initial state pairs (*ρ*_1_, *ρ*_2_). *σ*[*t*, *ρ*_1,2_(0)] is the rate of change of the trace distance, *σ*[*t*, *ρ*_1,2_(0)] = ∂_*t*_*D*[*ρ*_1_(*t*), *ρ*_2_(*t*)], with positive value indicating information flowing back to the system. The trace distance *D* measures the distinguishability between the two states, which is defined by 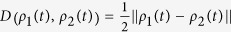, with 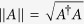 and 0 ≤ *D* ≤ 1. Here, for the optimal state pairs, the trace distance of the evolved states can be acquired by *D*_*t*_ = |*F*_*t*_|^9^. Thus, following ref. 24, we deliberately calculate the non-Markovianility as


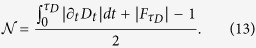


## Additional Information

**How to cite this article**: Wei, Y.-B. *et al.* Quantum speed limit and a signal of quantum criticality. *Sci. Rep.* **6**, 19308; doi: 10.1038/srep19308 (2016).
